# Deep Forest as a framework for a new class of machine-learning models

**DOI:** 10.1093/nsr/nwy151

**Published:** 2018-12-05

**Authors:** Lev V Utkin, Anna A Meldo, Andrei V Konstantinov

**Affiliations:** Laboratory of Neural Network Technology and Artificial Intelligence, Peter the Great St. Petersburg Polytechnic University, Russia

A new deep learning framework—the so-called Deep Forest (DF), proposed by Zhi-Hua Zhou and Ji Feng [1,2]—can be regarded as one of the important events of 2017 in machine learning, although it was unjustly unnoticed by a large number of researchers. The DF combines several ensemble-based methods, including Random Forests (RFs) and Stacking, into a structure that is similar to a multi-layer neural network, but each layer in the DF contains RFs instead of neurons. All advantages of DF are clearly discussed in [1–3]. In particular, DF is simple for training due to a very small number of hyper-parameters, it does not use back-propagation training and it outperforms many well-known methods, including deep neural networks, when there are only small-scale training data.

However, the main advantage of DF in our opinion is that it stimulates a relatively new trend in machine learning. Following pioneering work [1,2] and inspired by many virtues of the proposed model, new models improving DF are being proposed nowadays. That is why we suppose that Zhou and Feng introduced not a model, but a framework for developing new models and the whole direction in machine learning.

One of the virtues of neural networks is their flexibility of specifying the error or loss function depending on the data-processing task or a specific application. The variety of error functions allows the solving of a lot of machine-learning problems that are different from the standard classification, such as transfer learning and distance metric learning. Since a key element in the feature representation at every cascade level of DF is a probability class vector of every RF, which are computed by averaging the corresponding decision tree-class vectors across all trees in the RF; we can then specify a loss function by controlling the class vectors by means of weighing trees and computing weighted averages of the tree-class vectors, where weights are trained using the same training data. Moreover, we can apply some function of the tree-class vectors, which is implemented, for example, by a neural network. Modifications of DF with weights have demonstrated outperforming results [4] but, of course, they simultaneously complicate the models.

Another improvement of the original DF was proposed by Pang *et al.* [3] to significantly reduce the training and testing times of forests at each level. According to the improvement, training examples with high confidence (the maximum value of the estimated class vector) directly pass to the final stage rather than passing through all the levels. The improvement opens the door for developing new models with adaptive weighing of every training example at each cascade level, depending on its mean class vector at the previous level. The weighted examples are randomly chosen for training trees in accordance with their weights. The numerical experiments have shown that these models also provide outperformed results.

The idea of DF is a turning point that forces us to reconsider the nature of machine-learning approaches. An excellent exemplar of that is a model of multi-layered gradient-boosting decision trees [5].

## FUNDING

This work was supported by the Russian Science Foundation (18-11-00078).

## References

[1] Zhou Z-H , Feng J. Proceedings of the 26th International Joint Conference on Artificial Intelligence (IJCAI'17). Melbourne: AAAI Press, 2017, 3553–9.

[2] Zhou Z-H , Feng J. Natl Sci Rev 2019; 6: 74–86. 10.1093/nsr/nwy108 PMC8291612 34691833

[3] Pang M , Ting KM, Zhao P et al. Proceedings of the 18th IEEE International Conference on Data Mining (ICDM'18). Singapore: IEEE, 2018.

[4] Utkin LV , Ryabinin MA. Knowl-Based Syst 2018; 139: 13–22.

[5] Feng J , Yu Y, Zhou Z-H. Proceedings of the 32nd Conference on Neural Information Processing Systems (NIPS 2018). Montréal, Canada, 2018.

